# Novel Nanohybrids of Silver Particles on Clay Platelets for Inhibiting Silver-Resistant Bacteria

**DOI:** 10.1371/journal.pone.0021125

**Published:** 2011-06-17

**Authors:** Hong-Lin Su, Siou-Hong Lin, Jiun-Chiou Wei, I-Chuan Pao, Shu-Her Chiao, Chieh-Chen Huang, Shinn-Zong Lin, Jiang-Jen Lin

**Affiliations:** 1 Department of Life Sciences, National Chung Hsing University, Taichung, Taiwan; 2 Department of Materials Science and Engineering, National Chung Hsing University, Taichung, Taiwan; 3 Department of Physical Therapy, China Medical University, Taichung, Taiwan; 4 Department of Immunology, China Medical University, Taichung, Taiwan; 5 Center for Neuropsychiatry, China Medical University and Hospital, Taichung, Taiwan; 6 China Medical University Beigang Hospital, Yunlin, Taiwan; 7 Institute of Polymer Science and Engineering, National Taiwan University, Taipei, Taiwan; University of California, Merced, United States of America

## Abstract

We develop a novel nanohybrid showing a strong antibacterial activity on all of the tested pathogens, including methicillin-resistant *Staphylococcus auerus* and silver-resistant *E. coli*. The nanohybrid consists of silver nanoparticles (AgNPs) supported on 1 nm-thick silicate platelets (NSPs). The AgNP/NSP nanohybrid enables to encapsulate bacteria and triggers death signals from the cell membrane. The geographic shape of the NSPs concentrates AgNPs but impedes their penetration into attached cells, mitigating the detrimental effect of silver ion deposition in applied tissues. Moreover, the tightly tethered AgNPs on NSP surface achieve a stronger biocidal effect than silver nitrate, but bypassing Ag^+^ mechanism, on silver-resistant bacteria. This nanohybrid presents an effective and safe antimicrobial agent in a new perspective.

## Introduction

Soluble silver ion species are extensively used as antiseptics for controlling burn and eye infections. Silver ions can disrupt the bacterial cell wall, penetrate the cell and interfere with the physiological function of cell respiration and metabolites via binding to the thiol groups present in proteins [1], [2]. However, the frequent use of silver-ion agents has caused the appearance of notable silver resistant bacteria in hospitals, an emerging threat to public health [3], [4]. Similar to the mechanism of most heavy metal resistance systems, silver resistance is mediated by a plasmid- or chromosome-encoded Ag^+^ efflux pump [5]. Horizontal spreading of the plasmid can enhance the bacterial pathogenesis and resistance to antibiotics [3]. Hence, finding new antimicrobial agents and understanding the mechanism for inhibiting the silver-resistant bacteria are urgent research tasks.

Recent advances in nanotechnology have led the discovery of nanoscale inorganic materials that exhibit control over bacterial growth, such as silver-doped titanium oxide and silver nanoparticles (AgNPs) [6], [7], [8], [9]. However, the antimicrobial mechanism of AgNPs is not well clarified. Dissolved Ag^+^, material-cell contact and internalization of the nanoparticles could contribute to bacterial cell death [9], [10]. Although most studies [8], [11], [12] emphasize the importance of dissolved Ag^+^ on the antibacterial effect, several reports [13], [14], [15] claim that leaching Ag^+^ only cannot account for the AgNP cytotoxicity. In addition, the size and morphology of the nanoparticles also affect the biocidal performance [16], [17], [18]. Moreover, recent studies [19], [20], [21] and our previous report [22] suggest that the biocidal effectiveness of AgNPs primarily depends on the membrane disruption at the cell-material contacting surface, while the soluble Ag^+^ species leaching from AgNP is not the main thrust to the cytotoxicity. Based on the Ag^+^-independent bactericidal mechanism, AgNPs are considered to be effective for inhibiting silver-resistant bacteria. However, recent studies [16], [23], [24] revealed that pure AgNPs were ineffective at controlling silver-resistant bacteria, possibly because of the inherent tendency of AgNP agglomeration into Ag clusters and the consequent decrease in the particle/cell surface interaction.

To provide well-dispersed AgNPs in solution, a method of fabricating novel nanohybrids consisting of AgNPs immobilized on the surface of nanoscale silicate platelets (NSPs) was established. The high aspect-ratio NSPs were derived from layered silicate clay and in irregular polygonal shapes with an average platelet dimension of 80×80×1∼100×100×1 nm^3^ [25], [26]. NSP provides an extensive reacting surface for the formation of spherical AgNPs, synthesized by the *in situ* reduction of silver nitrate with methanol. Without any conventional organic stabilizers or sodium borohydride reducing agents, the naked silver nanoparticles were organic-free and fully reduced from Ag^+^ with a colloidal stability in water. This nanohybrid was examined for its efficacy in inhibiting the growth of both silver-sensitive and silver-resistant bacteria. The cell-surface interaction with the nanohybrid and the possible biocidal mechanism was also investigated.

## Results

### Synthesis of AgNP/NSP nanohybrid

**Figure 1:**
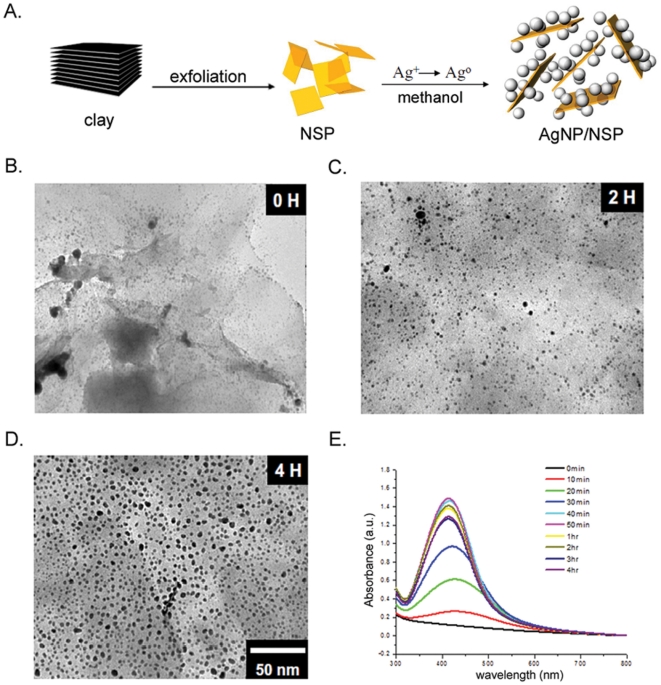
Monodispersed silver nanoparticles on NSPs. AgNP/NSP, synthesized at the equivalent ratio of Ag/CEC = 1.0, was fabricated by methanol reduction at 80°C. Diagram of the synthesis of the nanohybrid is illustrated (A). The surface structure of the AgNP/NSP was revealed by the FE-SEM (B-D). The absorbance at 420 nm kinetically revealed the reduced status of the AgNPs at the indicated times after methanol reduction (E).

The in situ reduction of silver nitrate in the presence of the silicate clay generated AgNP/NSP hybrids. The NSP material was prepared by exfoliating the Na^+^-MMT silicate clay, which involved the ionic exchange of the clay with a polyamine-salt exfoliating agent to randomize the layered structure [25]. The NSP material was characterized to have a fully exposed surface in comparison with that of the pristine MMT clay. The polyamine-salt was removed from the NSPs through a toluene and aqueous sodium hydroxide bi-phasic extraction. A single NSP possesses *ca.* 12,000∼20,000 ions at the dimension of 80×80∼100×100 nm^2^ (described in Materials and Methods). The 1-nm thick platelet shape of an NSP with a sodium ion at the cationic exchange capacity (CEC) of 1.2 mequiv/g [27] has a large surface area for associating silver nitrate and AgNP formation, following the methanol reduction (Fig. 1A).

After the 2 hr reduction of silver nitrate by methanol, small nanoparticles appeared on the NSP surface (Fig. 1B) when an equal molar ratio of Ag^+^ and CEC or the weight ratio of Ag^+^ to NSP of 7/93 was used (Fig. 1C). The stable AgNPs on NSP were generated after a 4 hr reduction and showed spherical shape with a 6.6±2.7 nm diameter, estimated from 200 AgNPs in Fig. 1D. The AgNPs were fully reduced from silver nitrate and colloidal stable in water, evidenced by the UV absorption at 414 nm (Fig. 1E). UV-Vis result revealed a reduced intensity of UV absorbance and a slightly shifted surface plasmon to a longer wavelength after 50 min reaction (Fig. 1E), potentially due to the enlarged particle size over 3.5 nm [28]. This phenomenon is also observed for the fabricated AgNPs with other clay, such as montmorillonite [19] or laponite [29].

Further tests on the dissolved Ag^+^ in solution showed only 356 ppb in 1.0 wt% AgNP/NSP solution. Adding nitric acid to convert the free Ag^0^ to Ag^+^ in solution resulted in the similar concentration of silver ion (395 ppb) by ICP-MS analysis, indicating that very few Ag^0^ particles shell off the NSP carrier. Because the nanohybrid was prepared without using any organic dispersant, the generated AgNP was considered to be naked surface and free of organic wrapping. These results demonstrate that the NSP is a suitable dispersing agent to fabricate fine inorganic nanoparticles.

### Antimicrobial activity of AgNP/NSP

**Figure 2:**
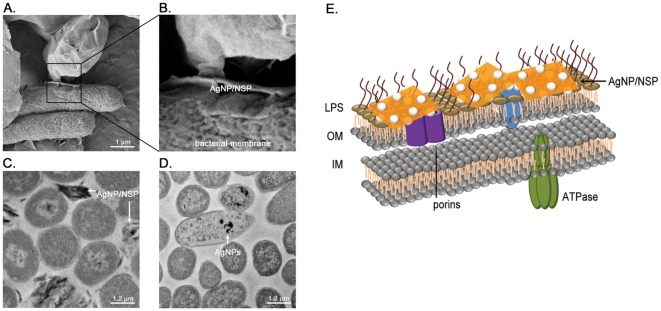
Material distribution in AgNP/NSP-treated *E. coli*. The *E. coli* were treated with 0.1 wt% AgNP/NSP (A, B, C) or free AgNPs (D) alone for 4 hr. The cell-nanomaterial complexes were examined using FE-SEM (A, B) and TEM (C, D). AgNP alone is used as a positive control for the nanomaterial accumulation in cells (D). Representive Diagram of the nanohybrid-bacteria interaction is illustrated (E). LPS, lipopolysaccharide, OM, outer membrane, IM, inner membrane.

**Figure 3:**
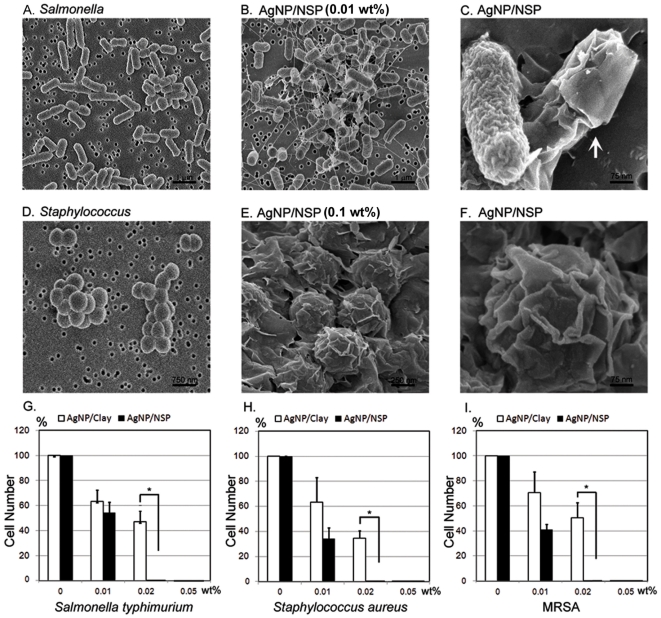
Material-cell interaction and antimicrobial activity of AgNP/NSP. *Salmonella typhimurium* (A–CD) and *Staphylococcus aureus* (D–FG) were treated with 0.01 wt% (B, C) and 0.1 wt% AgNP/NSP (E, F), respectively, for 6 hr at 37°C. The cell-nanomaterial complexes were examined using FE-SEM at 80 kV. The arrow indicates the nanohybrid-encapsulated bacteria (C). *S. typhimurium* (G), *S. aureus* (H) and methicillin-resistant *S. aureus* (MRSA) (I) were cultured on AgNP/NSP-containing LB agar and the growth inhibition was estimated by the colony formation at 14–16 hr at 37°C. *, p-values<0.05, Student's *t*-test.

The close-up images of AgNP/NSP-coated *E. coli* under FE-SEM (Fig. 2A and 2B) and TEM (Fig. 2C) revealed that all the observed NSP wrapped the bacteria and no AgNP is found in the cell body of *E. coli* (n = 595) (Fig. 2C). In contrast, when the bacteria were treated with 120 µM free AgNPs for 4 hr (Fig. 2D), AgNPs was found on the membrane or in the cells of E. coli (20% of counted cells, n = 1728). These observations suggest that the high electrostatic affinity of NSP may facilitate high adherence onto the bacterial surface and facilitate AgNP-cell interaction, causing local membrane damage (illustrated in Fig. 2E). The antibacterial potency was evaluated and compared to our previous findings for AgNP/Clay [22]. While treating *Salmonella typhimurium* with 0.01 wt% AgNP/NSP (7/93 by weight ratio) for 4 hr, the apparent clustering of bacteria was observed. This clustering was potentially caused by bridging the bacteria through the nanohybrid (Fig. 3A and 3B). Most applied nanohybrid attached to and encapsulated the surface of bacteria (Fig. 3C, the arrow). At a higher dosage of AgNP/NSP (0.1 wt%), *Staphylococcus aureus* were wrapped with the nanohybrids (Fig. 3E and 3F), but none of the bacterial membrane was found to be penetrated by the platelets. These observations suggest that most nanohybrids have intensive attracting forces to adhere on surface, which might interfere with the bacterial growth through a physical trapping mechanism. To determine the effectiveness of the biocidal activity, 1×10^5^ bacterial cells, such as the *Salmonella* (Gram negative) and *Staphylococcus* (Gram positive), were plated on the AgNP/NSP-containing LB agar. To determine the minimal inhibitory concentration (MIC), the complete inhibition of cell growth of tested bacterial strains was achieved at 0.02 wt% AgNP/NSP (Fig. 3G and 3H). These results were an improvement over the previous report using AgNP/Clay, in which 0.05 wt% material was required [22]. Here, the difference between the high aspect-ratio NSP and the pristine layered clay for the bacterial surface interaction is noteworthy.

**Figure 4:**
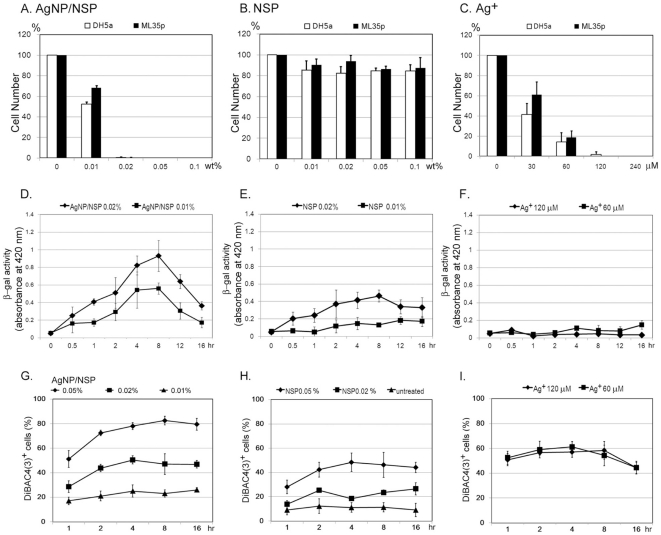
Disrupted membrane permeability and polarity by AgNP/NSP. Approximately 1×10^5^ *E coli* DH5α and ML35p strains were spread on NSP (A), AgNP/NSP (B) or silver nitrate (C) containing LB agars, where nanohybrid materials were mixed with at the indicated concentrations. These colonies were counted after overnight incubation at 37°C. After treating *E. coli* ML35p with the nanomaterials (D, E) or silver nitrate (F) in LB broth, the activity of the released intracellular β-galactosidase in the supernatant was kinetically determined by reacting with ONPG and quantitatively measured with spectrophotometry at 420 nm. The number of fluorescent DiBAC_4_(3)^+^ cells in NSP- (G), AgNP/NSP- (H) or silver nitrate- (I) treated *E. coli* DH5α were determined by FACS analysis.

Particularly, as the wild type *S. aureus* and the antibiotic-resistant MRSA showed the same vulnerability to the nanohybrid (Fig. 3I), other infectious pathogen, such as *Escherichia coli*, all showed complete inhibition on the 0.02 wt% AgNP/NSP-containing agar (Fig. 4A). The antibacterial performance of the nanohybrid illustrates the highly potent and general biocidal activity on all tested bacteria through the mechanism of physical adhesion to the bacterial cell.

### Antimicrobial mechanism of AgNP/NSP and Ag^+^

To discriminate the individual roles of NSP and AgNP on inhibiting bacterial growth, both NSP and AgNP/NSP were investigated for their antibacterial potencies on LB agar. We found that the growth of *E. coli* was significantly inhibited by the AgNP/NSP (Fig. 4A), but not NSP (Fig. 4B), indicating that the AgNP is the predominant factor governing antibacterial activity. The integrity of the cell membrane was first explored to understand the biocidal effect of the AgNP/NSP by determining the release of intracellular β-galactosidase, an indicator for the inner membrane leakage of bacteria [30]. After incubating with 0.01 wt% or 0.02 wt% AgNP/NSP, *E. coli* ML35p released a significant amount of intracellular β-galactosidase into the culture medium during the first 2 hr reaction (p<0.05 in both, Fig. 4D). The highest extracellular enzyme activity was detected after the 8 hr post-treatment and then the activity gradually declined. Notably, the treatment with 0.02 wt% NSP alone also resulted in moderate damage of the bacterial membrane at 4 hr and 8 hr in reference to the untreated control (p<0.05, Fig. 4E).

The membrane disruption could decrease the cell polarity and allow the retention of DiBAC_4_(3), a lipophilic anionic fluorophore and a quantitative indicator for the loss of transmembrane potential [31]. Treating *E. coli* with AgNP/NSP had resulted in a dose-dependent increased in the fraction of DiBAC_4_(3)^+^ cells (Fig. 4G). At 0.05 wt% and 0.02 wt% of nanohybrid, there was a conversion of 78.0±2.8% and 50.4±3.3% cells into DiBAC_4_(3)^+^ after a 4 hr incubation, respectively (p<0.05 in both). By comparison, treating with 0.05 wt% and 0.02 wt% NSPs alone triggered 48.4±7.6% (p<0.05) and 18.5±0.7% fluorescent cells observed, respectively (Fig. 4H). Taken together, these results demonstrated that treating AgNP/NSP with bacteria severely disrupts the inner membrane integrity (Fig. 4D and 4G). The support material of NSP has a low antimicrobial effect (Fig. 4B) but can trigger a transient and reversible disturbance of membrane permeability (Fig. 4E and 4H), which is potentially caused by the disorder of the lipopolysaccharide structure or loss of function of the outer membrane proteins on the encountered surface [32], [33].

The antibacterial mechanism involving the Ag^+^ was investigated, in order to compare with the above findings using the nanohybrids. The complete growth inhibition of *E. coli* was observed at a concentration of 120 µM silver nitrate, comparable to the potency of 0.02 wt% AgNP/NSP (containing 129.8 µM silver) (Fig. 4A and 4C). This result indicated that both the AgNP/NSP and Ag^+^ exhibited a similar MIC and biocidal activity on *E. coli* at the same silver concentration. Notably, upon treatment of Ag^+^ no β-galactosidase activity was detected using the membrane permeability assay (Fig. 4F), perhaps because of the rapid enzyme inactivation by silver nitrate in solution. However, Ag^+^ indeed caused the membrane damage and loss of transmembrane potential, evidenced by the intracellular staining of ionic voltage-sensitive DiBAC_4_(3) (Fig. 4I). Unexpectedly, the doses of 60, 120 and 300 µM silver nitrate all steered 50–60% DiBAC_4_(3)^+^ cells (Fig. 4I), distinctly different from the time and dose-dependent results obtained when using AgNP/NSP (Fig. 4G).

Experimental evidence of the complete reduction of Ag^+^ to Ag^0^ in the synthesis and low dissolvability of the AgNPs in solution suggested that the cytotoxicity of the nanohybrid may not be caused by the released Ag^+^. Furthermore, the supernatant of 1 wt% AgNP/NSP had shown no antibacterial activity even for the sample that had undergone a six-month period of storage. In addition,cell-released β-galactosidase remained functional in the supernatant of AgNP/NSP-treated cells (Fig. 4D). These results strongly indicated that the cytotoxicity of the AgNP/NSP was mainly dependent on the concentrated AgNPs on the NSP carrier, rather than on the soluble Ag^+^ species or free AgNPs in solution.

**Figure 5:**
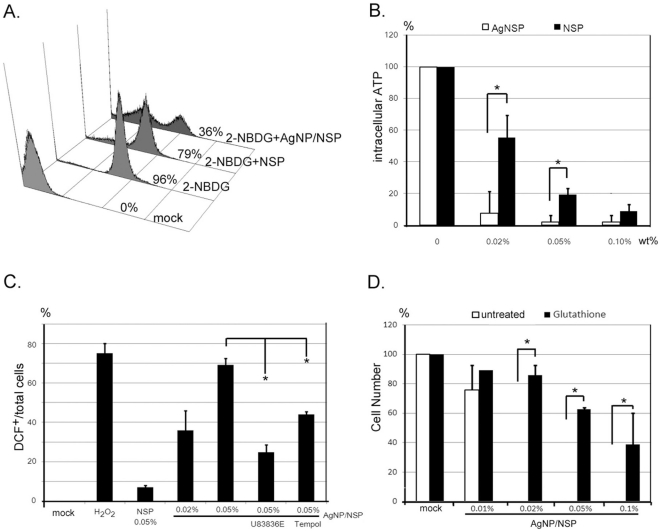
The biocidal pathway in AgNP/NSP treated cells. *E. coli* were cultured with NSPs or AgNP/NSP for 4 hr and subjected to biochemical analysis. The glucose uptake (A), ATP production (B) and intracellular ROS production (C) were determined by the incorporation of 2-NDBG, the luciferase-luciferin reaction and the H_2_DCF-DCF conversion, respectively. Ratios of 2-NDBG^+^ and DCF^+^ cells were determined by FACS analysis. For the ROS test, 1% H_2_O_2_ was used as positive control. U83836E, 50 µM. Tempol, 50 µM. Before the seeding of *E. coli*, 50 µl glutathione (10 mM) was applied on the AgNP/Clay containing agar plates (D). The number of bacterial colonies was counted and these data were obtained from three independent results. *, p-values<0.05, Student's *t*-test.

Severe membrane disruption may affect the nutrient uptake, electronic transport and ROS production. By feeding *E. coli* with a fluorescein-labelled glucose analog, 2-NBDG, almost all bacteria became green fluorescent (Fig. 5A). FACS analysis showed that the glucose uptake was dramatically impaired in AgNP/NSP-treated *E. coli* (36%) after the 4 hr incubation in comparison to those in nanomaterials-untreated control (96%) and NSP-treated cells (79%) (Fig. 5A). In addition, the intracellular ATP content was also significantly reduced in a dose-dependent manner at 4 hr post-treatment in AgNP/NSP-treated *E. coli*, compared to that of NSP-treated cells (Fig. 5B). Using the H2DCF staining and FACS analysis, robust ROS production was confirmed in 36.0±9.9% and 69.3±3.3% of the cells during the 4 hr incubation in which *E. coli* were treated with 0.02 wt% and 0.05 wt% nanohybrids, respectively (p<0.05 in both). In contrast, 0.05 wt% NSPs steered only a limited amount of DCF^+^ cells (7.0±0.8%, Fig. 5C). Furthermore, the pretreatment of ROS scavengers, such as lipid peroxidation inhibitor U83836E and a superoxide dismutase mimetic Tempol, limited the AgNP/NSP-steered ROS production (Fig. 5C). Glutathione, a well-known cellular anti-oxidant [34], successfully rescued the AgNP/NSP-induced cell death (Fig. 5D and Fig. S1), demonstrating the critical role of ROS in this biocidal pathway. These results emphasize that the AgNP/NSP nanohybrid, but not NSP alone, constitutively disrupts the integrity of membrane, impedes the nutrient uptake and produces detrimental free radicals on the encountered cells.

### Effectiveness of AgNP/NSP on silver-resistant bacteria

**Figure 6:**
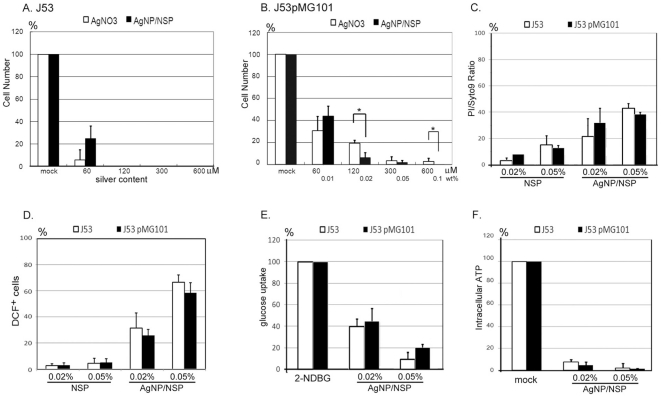
Inhibition of silver-resistant bacteria by AgNP/NSP. The cytotoxicity of silver nitrate and AgNP/NSP on *E. coli* J53 (A) or J53/pMG101 (B) were tested by colony growth on agar. Silver concentrations for 0.01, 0.02, 0.05 and 0.1 wt% AgNP/NSP were 64.9 µM, 129.8 µM, 324.5 µM and 648.9 µM, respectively. At 4 hr incubation with the nanomaterials, the ratios of fluorescent PI/Syto9 cells (C), DCF^+^ cells (D) and 2-NDBG^+^ cells (E) were determined by FACS analysis. The content of intracellular ATP was reflected by luciferase activity in the treated cells (F). The data represent triplicate (A, B) or duplicate experimental results (C–F). *, p-values<0.05, Student's *t*-test.

The introduction of NSP facilitates the cell-material interaction and provides a novel antimicrobial activity of AgNP/NSP, bypassing the Ag^+^-mediated intracellular protein/DNA denaturation. This unique biocidal property was further tested on bacteria with Ag^+^-resistance, which can be obtained by transfoming a pMG101 plasmid. Plasmid-free, parental *E. coli* J53 was silver-sensitive and showed complete growth inhibition on LB agar containing 120 µM silver nitrate or 0.02 wt% AgNP/NSP (Fig. 6A). Plasmid-transformed, silver-resistant J53/pMG101 rendered a 19.2±6.3% survival rate under 120 µM silver nitrate and yet 2.8±3.1% of the cells were detected even upon raising the silver concentration to 600 µM (MIC>600 µM). Compared to the 120 µM silver nitrate, 0.02 wt% AgNP/NSP (containing 129.8 µM silver) rendered only 5.9±4.0% surviving cells, indicating a significantly improved antimicrobial effect (student *t* test, p<0.05, Fig. 6B). In addition, the application of 0.1 wt% nanohybrids (containing 648.9 µM silver) completely blocked the growth of J53/pMG101, demonstrating the effectiveness of the AgNP/NSP on controlling silver-resistant bacteria (Fig. 6B).

Assuming that the difference between J53 and J53/pMG101 bacteria is only in the Ag^+^ channel system, both silver-sensitive and silver-resistant bacteria should have responded with the same sensitivity to AgNP/NSP. However, efficient controlling the growth of *E. Coli* J53/pMG101 required 0.1 wt% nanohybrid, which was five fold the dosage of the MIC in parental *E. Coli* J53. The higher MIC of the nanohybrid on *E. Coli* J53/pMG101 may be caused by elevated cellular antioxidant factors, deficiency of death-signal mediators or attenuated attacks on the membrane. To clarify these possibilities, the ratios of PI^+^- (Fig. 6C) and ROS-producing cells (Fig. 6D), reduction levels of intracellular ATP (Fig. 6E) and glucose uptake (Fig. 6F) were measured. These biochemical analyses all failed to show a significant difference in the AgNP/NSP-treated J53 and J53/pMG101 cells, indicating that the nature of the electrostatic affinity and attacking strength of the nanohybrid were conserved for both *E. coli* strains. In addition, producing intracellular ROS was also valid in silver-resistant strains (Fig. 6D), suggesting the propagation of AgNP/NSP-mediated death signal is not attenuated. Based on the glutathione rescue assay (Fig. 5D and Fig. S1) and the results shown in Fig. 6, overexpressed intracellular antioxidants in J53/pMG101 could account for the five fold MIC requirement of the nanohybrid to achieve complete growth inhibition of the silver-resistant bacteria.

## Discussion

We report the synthesis of homogenously dispersed AgNPs on silicate platelets without organic wrapping on the particle surface. The inorganic clay platelets served as a carrier for stabilizing the AgNPs without the inherent agglomeration by overcoming the ionic or van der Waals attraction among the nanoparticles. Compared to the six layers of stacked clay in our previous report [22], single-layered NSPs are fully exfoliated and show a higher CEC and full exposure of ionic charges. These characteristics enable the formation of smaller AgNPs (7 nm vs. 26 nm diameter of AgNP) and provide more cell-material contact surface, possibly accounting for the improved antibacterial effectiveness of this new nanohybrid.

Although high electrostatic activity of NSP can transiently disturb the cellular membrane integrity and cause a moderate alteration on the membrane permeability, NSP alone did not show significant biocidal activity in LB medium. Moreover, in contrast to NSP, AgNP/NSP exerts the significant growth reduction, dramatically decreased glucose uptake and ATP synthesis, and robust detrimental ROS production. These biochemical analyses emphasize that AgNP is the main thrust of AgNP/NSP, but not NSP, in bacterial interaction and microbial growth.

Enhancing the antimicrobial activity and reducing the nanotoxicity to mammalian cells will advance the clinical application of nanoparticles in medicine. Our newly synthesized AgNP/NSP exhibited a general and effective biocidal activity to all of the tested Gram-positive and Gram-negative bacteria herein, including MRSA. Notably, the silicate platelet NSP has shown none of obvious pathology in eukaryotes and rodents [25]. In addition, the nanohybrid contains very low concentrations of free Ag^+^ and AgNPs leaching from the nanohybrids. The geometric shape of high aspect-ratio NSP also impedes the penetration of immobilized AgNPs into mammalian cells. These characteristics help the applied nanohybrids to avoid deposition of detrimental silver in the body, illustrating the safety advantage of nanohybrid for clinical applications.

The newly developed nanohybrid exhibited general antibacterial activity, and stronger potency than silver ions, in controlling silver-resistant bacteria. The mechanistic understanding of the thrust of nanomaterials will bring forth the promise for efficient control of emerging public-threat pathogens.

## Materials and Methods

### Materials preparation of NSPs and AgNP/NSP

NSPs are prepared from a Na^+^ form of layered smectite clay, montmorillonite (Na^+^-MMT, from Nanocor), according to the exfoliation process reported previously [25]. The total surface area of 1 g Na^+^-MMT is about 750 m^2^/g [35]. The CEC is 1.2 meq/g [27], which is equivalent to 7.2×10^20^ monovalant cations per gram of MMT (CEC×Avogadro's number 6.02×10^23^). The surface area per monovalent exchangeable cation is (750×10^18^ nm^2^/g)/(1.2 meq/g×6.02×10^23^) = 1.03 nm^2^/ion. The surface area of each NSP is 80×80 to 100×100 nm^2^ [25], [26]. Therefore, both sides of NSP layer contain 12,427 (80×80×2/1.03)∼19,420 (100×100×2/1.03) cations approximately.

The procedures for preparing the AgNP/NSP nanohybrid are described in the following sentences. NSPs (0.3 g in 30 g water; 1.0 wt%) were dispersed in deionized water at 80°C and mechanically stirred for several hours, followed by the addition of an AgNO_3_ solution (0.061 g of 1.0 wt% in water) at an equal molar ratio of Ag^+^ to cationic exchange capacity (CEC). The process involved the replacement of Ag^+^ with Na^+^ counter ions on the clay surface and, consequently, reduction of the Ag^+^ by ethanol (18.05 ml). The reaction mixture was stirred vigorously and monitored by observing the color change from yellow to deep-red, indicating the reduction of Ag^+^ to Ag^0^. The UV absorption at 414 nm indicates the formation of Ag nanoparticles. The particle sizes were measured by field emission scanning electronic microscopy (FE-SEM, Zeiss EM 902A) at 80 kV. The size distribution was measured from an average of 100 individual particles.

The concentration of dissolved Ag^+^ in solution was determined by inductively coupled plasma mass spectrometry (ICP-MS, PE-SCIEX ELAN 6100 DRC). The supernatant of a 1.0 wt% AgNP/NSP in solution was collected after centrifugation at 16,000× g for 30 min. The ICP-MS analysis showed that the Ag^+^ concentration was 356 ppb. After adding 3% HNO_3_ to the supernatant to convert the free Ag^0^ to Ag^+^, the concentration changed to 395 ppb.

### Sources of bacteria

*Escherichia coli* (*E. coli*) strains DH5α, ML35, J53 and J53pMG101 were grown at 37°C and maintained on Luria-Bertani (LB) agar. The ML35 strain was obtained from the American Type Culture Collection (ATCC, cell number 42827). The J53 and J53/pMG101 stains were kindly provided by Prof. Chi-Ming Che at the University of Hong Kong and Prof. Anne Summers at the University of Georgia, USA. *Staphylococcus aureus* (*S. aureus*), including methicillin-resistant *S. aureus* (MRSA), were isolated from Taichung Veteran General Hospital. *Salmonella typhimurium* was provided from the Animal Technology Institute of Taiwan. Handling of the potentially hazardous bacteria, especially the MRSA and J53/pMG101 strains, was performed strictly according to the standard biosafety procedures of National Chung Hsing University.

### Bactericidal effect of nanomaterials

The bacteria were synchronized at the log phase of the growth curve (OD_600_ 0.4∼0.6; ∼10^9^ cfu/ml), and an aliquot of log-phase cells (∼10^5^ cells) were then reacted with nanomaterials in LB broth or on LB agar. The reduction of bacterial growth and the minimal inhibitory concentration (MIC) were determined by the number of colonies after an overnight incubation on LB agar at 37°C. The colony number of untreated cells is set as 100%.

### Evaluation of inner membrane permeability

*E. coli* ML35p, a pUC19 plasmid-transformed ML35 strain, is generally used for investigating cell membrane permeability because of its high expression levels of intracellular β-galactosidase and lack of lactose permease [30]. The cells were incubated to log-phase growth (OD_600_ 0.4–0.6) at 37°C with 1 mM isopropyl β-D-1-thiogalactopyranoside (IPTG), an allolactose analog, to induce the synthesis of the pUC19-encoded β-galactosidase. Cells were washed and resuspended in the inner membrane wash buffer (10 mM NaH_2_PO_4_, 0.1 M NaCl) before reacting with the testing nanomaterials. For the time-interval experiments, the reaction of bacteria with materials was set at 4°C in LB broth to provide a consistent cell number. At indicated times, the cells and materials were centrifuged at 3,000× g and the supernatants were collected and reacted with *ortho*-nitrophenyl-β-galactoside (ONPG) for 10 min, which provides a colorimetric index of the b-galactosidase activity. The samples were measured spectrophotometry at 420 nm (Paradigm, Beckman-Coulter).

### Alteration of bacterial membrane potential

DiBAC_4_(3) (Invitrogen, USA) was used as an indicator in the change of membrane potential [31]. After the reaction, the treated cells were labeled with DiBAC_4_(3) (10 µg/ml) for 30 min at room temperature. The quantitative measurement of DiBAC_4_(3)^+^ cells was determined by flow cytometry (FACScan, Becton-Dickinson).

### Glucose uptake assay

A cell-impermeable, fluorescent glucose analog, 2-NDBG (2-(N-(7-nitrobenz-2-oxa-1, 3-diazol-4-yl)amino)-2-deoxyglucose), was used to determine the nutrient uptake efficiency of the bacteria [36]. Cells in log-phase of growth (OD_600_ 0.4–0.6) were incubated with the testing material for 4 hr in LB broth. The cells were centrifuged and resuspended in a 2-NDBG (100 µM) phosphate buffered solution (PBS) for 1 hr at 37°C. The quantitative analysis of 2-NDBG^+^ cells was performed using flow cytometry (FACScan, Becton-Dickinson).

### Intracellular ATP determination

The intracellular ATP content was measured by the ATP determination kit (Molecular Probes, USA). Cells in log-phase growth were treated with the nanomaterials in 50 mM HEPES buffer (pH 7.0) containing 5 mM glucose at room temperature for 4 hr. The cells were lysed with 1% trichloroacetic acid in the presence of 2 mM EDTA. After a 30-min incubation at 4°C, the samples were neutralized with two volumes of 0.1 M Tris acetate (pH 7.8). The cell aliquots (5 µl each) were added to luciferase-luciferin reaction buffer (95 µl) in 96-well plates and their ATP contents were reflected by the intensity of luminescence (Paradigm, Beckman-Coulter). The exact amount of ATP in the experimental samples was calculated from the established standard curve.

### ROS detection and free radicals blocking assay

The bacteria in log-phase growth were treated with the nanomaterials for 4 hr in LB medium and then reacted with an intracellular ROS indicator, H_2_DCFDA (50 µM, 2′,-7′-dichlorofluorescin diacetate, Invitrogen) for 30 min at room temperature [22]. The quantitative evaluation of the ROS-producing cells was determined by flow cytometry (FACScan, Becton-Dickinson). Two ROS inhibitors, U83836E (50 µM, Tocris) and Tempol (50 µM, Tocris), were used to pretreat the *E. coli* for 1 hr before the cell-materials reaction. The Tempol/U83836E antioxidants were removed before the attack of AgNP/NSP. In a glutathione-rescued survival assay, glutathione (50 µl, 10 mM) was applied on a LB agar plate and then approximately 1×10^5^ cells were seeded onto the plates. After a 14 hr incubation at 37°C, colony formation units were manually counted.

### Statistical analysis

Each experiment was replicated two or three times and the data are presented as mean ± SEM (standard error of mean). A statistical analysis of the data was performed using Microsoft Excel. Differences were analyzed with Student's *t*-test and considered significant at p-values<0.05.

## Supporting Information

Figure S1 Growth rescue by the glutathione pretreatment for the AgNP/NSP-coated *E. coli*. The *E.coli* were first reacted with 200 µM glutathione for 1 hr at room temperature. The cells were concentrated by centrifuge at 13,000 rpm for 5 min and resupended in PBS containing 0.02 wt% AgNP/NSP for 4 hr. The cell surviving rate is estimated by the number of cell colonies on LB agar plates after 14 hr incubation at 37°C. *, p-values<0.05, Student's *t*-test.
(EPS)

## References

[1] Feng QL, Wu J, Chen GQ, Cui FZ, Kim TN (2000). A mechanistic study of the antibacterial effect of silver ions on Escherichia coli and Staphylococcus aureus.. J Biomed Mater Res.

[2] Jung WK, Koo HC, Kim KW, Shin S, Kim SH (2008). Antibacterial activity and mechanism of action of the silver ion in Staphylococcus aureus and Escherichia coli.. Appl Environ Microbiol.

[3] Silver S (2003). Bacterial silver resistance: molecular biology and uses and misuses of silver compounds.. FEMS Microbiol Rev.

[4] Woods EJ, Cochrane CA, Percival SL (2009). Prevalence of silver resistance genes in bacteria isolated from human and horse wounds.. Vet Microbiol.

[5] Gupta A, Matsui K, Lo JF, Silver S (1999). Molecular basis for resistance to silver cations in Salmonella.. Nat Med.

[6] Wong MS, Sun DS, Chang HH (2010). Bactericidal performance of visible-light responsive titania photocatalyst with silver nanostructures.. PLoS One.

[7] Martinez-Gutierrez F, Olive PL, Banuelos A, Orrantia E, Nino N (2010). Synthesis, characterization, and evaluation of antimicrobial and cytotoxic effect of silver and titanium nanoparticles.. Nanomedicine.

[8] Gunawan C, Teoh WY, Marquis CP, Lifia J, Amal R (2009). Reversible antimicrobial photoswitching in nanosilver.. Small.

[9] Kim JS, Kuk E, Yu KN, Kim JH, Park SJ (2007). Antimicrobial effects of silver nanoparticles.. Nanomedicine.

[10] Baker C, Pradhan A, Pakstis L, Pochan DJ, Shah SI (2005). Synthesis and antibacterial properties of silver nanoparticles.. J Nanosci Nanotechnol.

[11] Lee D, Cohen RE, Rubner MF (2005). Antibacterial properties of Ag nanoparticle loaded multilayers and formation of magnetically directed antibacterial microparticles.. Langmuir.

[12] Liu J, Sonshine DA, Shervani S, Hurt RH (2010). Controlled Release of Biologically Active Silver from Nanosilver Surfaces.. ACS Nano.

[13] Wei L, Tang J, Zhang Z, Chen Y, Zhou G (2010). Investigation of the cytotoxicity mechanism of silver nanoparticles in vitro.. Biomed Mater.

[14] Navarro E, Piccapietra F, Wagner B, Marconi F, Kaegi R (2008). Toxicity of silver nanoparticles to Chlamydomonas reinhardtii.. Environ Sci Technol.

[15] Wigginton NS, Titta A, Piccapietra F, Dobias J, Nesatyy VJ (2010). Binding of silver nanoparticles to bacterial proteins depends on surface modifications and inhibits enzymatic activity.. Environ Sci Technol.

[16] Samberg ME, Orndorff PE, Monteiro-Riviere NA (2010). Antibacterial efficacy of silver nanoparticles of different sizes, surface conditions and synthesis methods.. Nanotoxicology.

[17] Liu W, Wu Y, Wang C, Li HC, Wang T (2010). Impact of silver nanoparticles on human cells: effect of particle size.. Nanotoxicology.

[18] Pal S, Tak YK, Song JM (2007). Does the antibacterial activity of silver nanoparticles depend on the shape of the nanoparticle? A study of the Gram-negative bacterium Escherichia coli.. Appl Environ Microbiol.

[19] Miyoshi H, Ohno H, Sakai K, Okamura N, Kourai H (2010). Characterization and photochemical and antibacterial properties of highly stable silver nanoparticles prepared on montmorillonite clay in n-hexanol.. J Colloid Interface Sci.

[20] Kim S, Choi JE, Choi J, Chung KH, Park K (2009). Oxidative stress-dependent toxicity of silver nanoparticles in human hepatoma cells.. Toxicol In Vitro.

[21] Kawata K, Osawa M, Okabe S (2009). In vitro toxicity of silver nanoparticles at noncytotoxic doses to HepG2 human hepatoma cells.. Environ Sci Technol.

[22] Su HL, Chou CC, Hung DJ, Lin SH, Pao IC (2009). The disruption of bacterial membrane integrity through ROS generation induced by nanohybrids of silver and clay.. Biomaterials.

[23] Lok CN, Ho CM, Chen R, He QY, Yu WY (2007). Silver nanoparticles: partial oxidation and antibacterial activities.. J Biol Inorg Chem.

[24] Hsu SH, Tseng HJ, Lin YC (2010). The biocompatibility and antibacterial properties of waterborne polyurethane-silver nanocomposites.. Biomaterials.

[25] Li PR, Wei JC, Chiu YF, Su HL, Peng FC (2010). Evaluation on cytotoxicity and genotoxicity of the exfoliated silicate nanoclay.. ACS Appl Mater Interfaces.

[26] Hsu SH, Tseng HJ, Hung HS, Wang MC, Hung CH (2009). Antimicrobial activities and cellular responses to natural silicate clays and derivatives modified by cationic alkylamine salts.. ACS Appl Mater Interfaces.

[27] Alexandre M, Dubois P (2000). Polymer-layered silicate nanocomposites: preparation, properties and uses of a new class of materials.. Mater Sci Eng.

[28] Kreibig U, Fragstein CVZ (1969). The limitation of electron mean free path in small silver particles.. Phys.

[29] Liu J, Lee J-B, Kim D-H, Kim Y (2007). Preparation of high concentration of silver colloidal nanoparticles in layered laponite sol.. Colloids Surf A.

[30] Epand RF, Mowery BP, Lee SE, Stahl SS, Lehrer RI (2008). Dual mechanism of bacterial lethality for a cationic sequence-random copolymer that mimics host-defense antimicrobial peptides.. J Mol Biol.

[31] Amor KB, Breeuwer P, Verbaarschot P, Rombouts FM, Akkermans AD (2002). Multiparametric flow cytometry and cell sorting for the assessment of viable, injured, and dead bifidobacterium cells during bile salt stress.. Appl Environ Microbiol.

[32] Abu-Lail NI, Camesano TA (2003). Role of lipopolysaccharides in the adhesion, retention, and transport of Escherichia coli JM109.. Environ Sci Technol.

[33] Hammer MU, Brauser A, Olak C, Brezesinski G, Goldmann T (2010). Lipopolysaccharide interaction is decisive for the activity of the antimicrobial peptide NK-2 against Escherichia coli and Proteus mirabilis.. Biochem J.

[34] Ross D (1988). Glutathione, free radicals and chemotherapeutic agents. Mechanisms of free-radical induced toxicity and glutathione-dependent protection.. Pharmacol Ther.

[35] Helmy AK, Ferreiro EA, de Bussetti SG (1999). Surface Area Evaluation of Montmorillonite.. J Colloid Interface Sci.

[36] Mironova R, Niwa T, Hayashi H, Dimitrova R, Ivanov I (2001). Evidence for non-enzymatic glycosylation in Escherichia coli.. Mol Microbiol.

